# IDO1 and inflammatory neovascularization: bringing new blood to tumor-promoting inflammation

**DOI:** 10.3389/fonc.2023.1165298

**Published:** 2023-04-27

**Authors:** Alexander J. Muller, Arpita Mondal, Souvik Dey, George C. Prendergast

**Affiliations:** ^1^ Lankenau Institute for Medical Research, Wynnewood, PA, United States; ^2^ Sidney Kimmel Cancer Center, Thomas Jefferson University, Philadelphia, PA, United States; ^3^ Arbutus Biopharma, Inc., Warminster, PA, United States; ^4^ Wuxi Advanced Therapeutics, Inc., Philadelphia, PA, United States; ^5^ Department of Pathology, Anatomy and Cell Biology, Thomas Jefferson University, Philadelphia, PA, United States

**Keywords:** cancer immunology, tumor-promoting inflammation, immunometabolism, tryptophan, immune tolerance, neovascularization, indoleamine 2, 3-dioxygenase (IDO)

## Abstract

In parallel with the genetic and epigenetic changes that accumulate in tumor cells, chronic tumor-promoting inflammation establishes a local microenvironment that fosters the development of malignancy. While knowledge of the specific factors that distinguish tumor-promoting from non-tumor-promoting inflammation remains inchoate, nevertheless, as highlighted in this series on the ‘Hallmarks of Cancer’, it is clear that tumor-promoting inflammation is essential to neoplasia and metastatic progression making identification of specific factors critical. Studies of immunometabolism and inflamometabolism have revealed a role for the tryptophan catabolizing enzyme IDO1 as a core element in tumor-promoting inflammation. At one level, IDO1 expression promotes immune tolerance to tumor antigens, thereby helping tumors evade adaptive immune control. Additionally, recent findings indicate that IDO1 also promotes tumor neovascularization by subverting local innate immunity. This newly recognized function for IDO1 is mediated by a unique myeloid cell population termed IDVCs (IDO1-dependent vascularizing cells). Initially identified in metastatic lesions, IDVCs may exert broader effects on pathologic neovascularization in various disease settings. Mechanistically, induction of IDO1 expression in IDVCs by the inflammatory cytokine IFNγ blocks the antagonistic effect of IFNγ on neovascularization by stimulating the expression of IL6, a powerful pro-angiogenic cytokine. By contributing to vascular access, this newly ascribed function for IDO1 aligns with its involvement in other cancer hallmark functionalities, (tumor-promoting inflammation, immune escape, altered cellular metabolism, metastasis), which may stem from an underlying role in normal physiological functions such as wound healing and pregnancy. Understanding the nuances of how IDO1 involvement in these cancer hallmark functionalities varies between different tumor settings will be crucial to the future development of successful IDO1-directed therapies.

## Introduction

Inflammation, which is rooted in the evolutionarily ancient process of adaptively responding to compromised tissue integrity to restore homeostasis, involves multi-faceted and dynamic interactions between resident and infiltrating immune cells and stromal cells orchestrated to eliminate the cause of an injury, clear out necrotic cells and damaged tissue, and initiate tissue repair (1). An acute inflammatory response is self-limiting, proceeding in phases from initiation to resolution. However, when inflammation is not resolved and becomes chronic, it can contribute to a variety of pathologies including the promotion of malignancy (2). While clinical recognition of an apparent pathophysiological link between inflammation and cancer has a long history stretching back to the early 19^th^ century (3), cancer genetics in the modern era instead embraced the perception of cancer as essentially a cell intrinsic disease driven by somatic mutations within the nascent tumor cells, with little consideration that the extrinsic environment might have a determinative role (4). As such, the first rendition of “The Hallmarks of Cancer” published in 2000, focused exclusively on six essential functional capabilities that somatic cells must acquire to effectively form tumors, all of which described cell-intrinsic attributes with the exception of acknowledging the importance of establishing an external blood supply to support tumor outgrowth (5). The field shifted rapidly within the next decade, however, so that by the subsequent “Hallmarks of Cancer: The Next Generation”, recognition of tumor-promoting inflammation was included as an ‘enabling characteristic’ (6). It is now firmly established in cancer biology that the TME (tumor microenvironment), including stromal fibroblasts, vascular cells and immune cells together with cancer cells, comprise an integrated network that contextually define an individual tumor, and that inflammation has a vital role in shaping the TME (7).

Due to its complex and dynamic nature, inflammation can have pro-tumorigenic or anti-tumorigenic consequences, and interventions that can shift this balance have already proven to be beneficial. Specifically, an inflammatory environment is a positive prognostic indicator for responsiveness to immune checkpoint therapy with CTLA4 (cytotoxic T-lymphocyte-associated protein 4; CD152) or PD1/PDL1 (programmed cell death 1/programmed cell death ligand 1; CD279/CD274) targeting antibodies because these agents remove key components of the immune blockade that suppress tumor-directed effector T cells from mounting an anti-tumor response (8). Conversely, inflammation can also be modified to accentuate its tumor-promoting capacity. This review focuses on the IDO1 enzyme as one important determinant of a tumor-promoting inflammatory environment. In particular, we highlight how IDO1 acts as an integrative node for multiple ‘cancer hallmarks’, shaping a tumor-promoting inflammatory environment through altered cellular metabolism that promotes both immune escape and neovascularization.

## IDO1 studies

### IDO1 and immunologic tolerance, from embryos to tumors

IDO1 (indoleamine 2,3-dioxygenase 1) is a heme-containing enzyme that initiates the catabolism of tryptophan as the first step of the kynurenine pathway, which can lead to the *de novo* production of NAD and is separate from the serotonin pathway (9). However, it is a second, evolutionarily distinct enzyme, TDO2 (tryptophan 2,3-dioxygenase), catalyzing the same reaction as IDO1 that is responsible for maintaining tryptophan homeostasis (9–11). TDO2 is expressed predominantly in the liver although some biological activity has been implicated in the brain and aberrant TDO2 upregulation has been detected in various malignancies (9, 11). In contrast, IDO1 is expressed in a variety of tissues other than liver, exhibits a broader substrate specificity than TDO2 (including the D-isomer of tryptophan and various indoles), and is not responsive to circulating tryptophan levels (12). Instead, IDO1 is principally induced by the Th1-associated, inflammatory cytokine IFNγ (interferon-gamma) (13). The discovery that targeting IDO1 could subvert the protection from maternal immunity that a hemiallogeneic fetus requires for successful gestation (14) was followed by demonstrations that inhibiting IDO1 could relieve T cell-directed immunological suppression in cancer (15–17). IDO1 was thus implicated in the establishment of peripheral immune tolerance.

IDO1 is not expressed directly in T cells, but its expression in APCs (antigen presenting cells), particularly in certain DC (dendritic cell) subsets, has been linked to both the direct suppression of Teff (effector T) cells and the enhancement of Treg (regulatory T) cell activity. The effect on Tregs is mediated both by inducing Treg development from naïve T cells and by increasing the suppressive activity of existing Tregs. Detailed reviews delving into the different ways that IDO1 has been found to exert tolerogenic suppression of Teff cell function have been published elsewhere (18, 19). One longstanding debate, over the course of these investigations, has been over the relative importance of tryptophan depletion versus tryptophan catabolites as the mechanistic basis for exerting tolerogenic control. Tryptophan depletion has been linked to the activation of GCN2 (general control nonderepressible 2), one of four serine kinases that phosphorylates eIF2alpha (α-subunit of initiation factor 2) to initiate the ISR (integrated stress response) (20). Biologically, it was reported that ablating GCN2 expression in T cells rendered them nonresponsive to the suppressive influence of IDO1 (21). It has also been proposed that IDO1-mediated tryptophan depletion may exert effects by suppressing activation of mTOR (mammalian target of rapamycin) (22). Alternatively, while biological effects have been ascribed to a number of catabolites in the IDO1-initiated kynurenine pathway, most attention is currently focused on the ability of kynurenine itself to act as an AHR (aryl hydrocarbon receptor) ligand (23, 24). Kynurenine signaling through AHR functions as an oncometabolite through transcriptional activation of pro-growth genes in tumor cells and inhibition of T cell activity (25). The complex web of pro-tolerogenic effects that have been ascribed to IDO1 suggests that multiple mechanisms could be at play, and some conceptual models have tried to integrate both depletion and catabolites as contributing factors (26). Overall, however, the preponderance of evidence favors the role of catabolites, with the main objection to depletion being that circulating levels of tryptophan are simply too high for IDO1-expression by APCs to exert a physiologically relevant environmental impact through this mechanism of action (27). Evidence that direct depletion of kynurenine *in vivo* using PEGylated kynurenase recapitulates the anti-tumor effects of targeting IDO1, also bolsters the argument in favor of catabolites, and kynurenine in particular, as the mechanism of action through which IDO1 imposes T cell tolerance (28).

### IDO1 is a tumor-promoting modifier of inflammation

The enhanced anti-tumor immune responses achieved with IDO1 inhibitor treatment suggested that induction of IDO1 activity may be selected for as a means for tumors to escape immune surveillance (29). To explore this hypothesis, studies were performed using the classic, two-stage skin carcinogenesis model in which mice are exposed to a single application of the carcinogen DMBA (7,12-Dimethylbenz[*a*]anthracene) followed by successive applications of the tumor-promoting agent TPA (12-O-Tetradecanoylphorbol 13-acetate; aka PMA, Phorbol 12-myristate 13-acetate). TPA is a tumor-promoting compound that, following initiation with the mutagen DMBA, accentuates tumorigenesis as assessed by the formation of premalignant lesions, referred to as papillomas, on the skin. Without an initiating insult, TPA does not elicit tumor outgrowth but rather produces a localized inflammatory response at the site of exposure (30). Notably, TPA treatment was found to stimulate IDO1 induction within dendritic cells in the regional lymph nodes, and genetic ablation of IDO1 substantially reduced the number of papillomas that formed in DMBA/TPA treated mice relative to their WT (wild type) counterparts (31). While papilloma development was significantly impaired by IDO1 gene deletion, this effect was unrelated to any discernable reduction in the degree of inflammation elicited by TPA treatment (32). Furthermore, when TPA was eliminated from the carcinogenesis process by instead eliciting tumors though multiple rounds of DMBA application, IDO1 gene deletion had no demonstrable impact on the incidence of papilloma formation. Thus, rather than acting as a fundamental driver of either inflammation or tumorigenesis, IDO1 acted as a modifier of the inflammatory milieu, changing its metabolic character and rendering it more supportive of tumor development (33).

Of note, the administration of TPA alone was sufficient to induce IDO1 in DCs within the regional lymph nodes (31). In previous work, it was determined that loss of the BIN1 (bridging integrator 1) tumor suppressor resulted in dysregulated induction of IDO1 in oncogenically transformed skin fibroblasts thereby facilitating immune escape when the cells were grafted into immune competent hosts (16). These results were consistent with IDO1 induction in tumor cells being a later immune escape event acquired during malignant progression (34). However, the DMBA/TPA carcinogenesis data suggest that local inflammatory conditions can result in the induction of IDO1 in immune cells even in the absence of any pre-existing carcinogenic insult, as DMBA administration is not required for IDO1 induction in the DMBA/TPA model. In this case, rather than being acquired under selective pressure, IDO1 induction precedes tumor initiation, priming the inflammatory TME to enable nascent tumors to bypass the elimination and equilibrium phases of immune surveillance and proceed directly to the escape phase. Taken together, these data offered genetic evidence defining IDO1 as a pro-tumorigenic modifier of the inflammatory milieu.

### IDO1 is coupled to pathogenic inflammatory neovascularization

To expand on the role of IDO1 in tumor progression, it was of interest to explore how IDO1 might contribute to spontaneous tumorigenesis that was not elicited by chemically-driven tumor initiation/promotion. For these studies, lung cancer and metastases models were selected because IDO1 was known to be highly inducible in lung tissue (35). An autochthonous lung cancer model has been developed in which tumor development is initiated by infecting the lungs of transgenic mice harboring latent oncogenic *Kras* (Kirsten rat sarcoma viral oncogene homolog) with an activating adenovirus vector-expressing Cre recombinase. This system affords control over the timing and breadth of oncogenic initiation in lung tissue, based on when and at what MOI (multiplicity of infection) the *Kras*-activating adenovirus is administered. Tumors that develop in these mice exhibit many characteristics of human NSCLC (non-small cell lung carcinoma) (36). In this model system, genetic loss of IDO1 caused a substantial delay in tumor outgrowth (37). Similarly, in a pulmonary metastasis model in which orthotopically engrafted 4T1 breast adenocarcinoma cells metastasize spontaneously to the lungs, *Ido1* ^-/-^ (*Ido1*-nullyzygous) animals exhibited significantly delayed pulmonary tumor outgrowth relative to WT animals. In this model, the engrafted 4T1 tumor cells have wild type *Ido1* alleles, indicating that metastasis resistance is caused by loss of IDO1 in host cells rather than in tumor cells. Attenuated elevation of the inflammatory cytokine IL6 was observed in both the autochthonous and metastatic tumor models, suggesting a manner by which IDO1 might orchestrate a pro-tumorigenic, inflammatory microenvironment. Consistent with this interpretation, ectopic expression of IL6 in engrafted 4T1 cells overcame the impairment in lung metastasis outgrowth educed in *Ido1* ^-/-^ mice.

One striking finding during the course of this work arose from observations from CT (computed tomography) scans employed to monitor the impact of IDO1 loss on lung tumor development. Specifically, these scans revealed that *Ido1* ^-/-^ mice had a baseline reduction in lung vascularization (37). In like manner, 4T1 lung metastases that formed in *Ido1* ^-/-^ mice also exhibited reduced blood vessel density relative to metastases formed in WT animals (38). Together these observations suggested that impaired neovascularization might contribute to the delayed lung metastasis outgrowth observed in *Ido1* ^-/-^ mice. One reported attribute of IFNγ, the principal inducer of IDO1 expression, is its ability to curtail neovascularization in tumors (39–41). In mice in which both IFNγ and IDO1 were genetically deleted, the levels of 4T1 lung metastasis neovascularization were restored to WT levels, confirming that the observed reduction in blood vessel density in *Ido1* ^-/-^ mice was attributable to IFNγ. Correspondingly, the delay in tumor outgrowth associated with IDO1 loss was likewise overcome by the concomitant loss of IFNγ. Together, these findings established a novel aspect of IDO1’s function in cancer, namely, its ability to contravene an anti-tumorigenic, IFNγ-mediated inflammatory environment that restricts new blood vessel formation in tumors.

**Figure 1:**
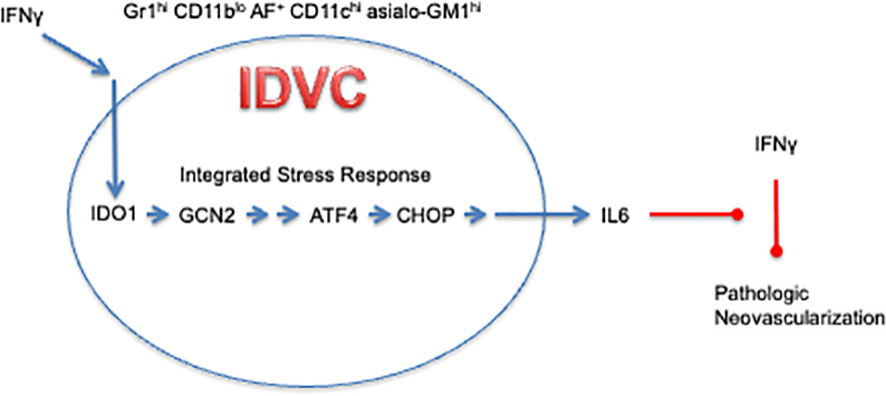
IDO1-Dependent Vascularizing Cells contravene the ability of IFNγ to regress neovascularization through IDO1-mediated induction of IL6.

An essential mediator of this pro-tumorigenic effect of IDO1 was found to be IL6. In contrast to IFNγ, which is generally considered to be anti-tumorigenic (42), IL6 is an inflammatory cytokine with pro-tumorigenic properties including the ability to support angiogenesis (43). Consistent with data that ectopic IL6 expression could restore 4T1 lung metastasis growth in *Ido1* ^-/-^ mice, genetic loss of IL6 resulted in impaired 4T1 metastasis outgrowth and reduced neovascularization; moreover, both of these effects were obviated by concomitant loss of IFNγ in double knockout *Il6* ^-/-^ *Ifng* ^-/-^ mice (38). Together, these results yielded a conceptual model whereby IDO1 acts as a regulatory node at the interface between IFNγ and IL6, shifting the inflammatory microenvironment to a tumor-promoting state by preventing the ability of IFNγ to pare back the tumor neovasculature (**Figure 1**). This differentiates IDO1 from pro-angiogenic factors such as VEGF (vascular endothelial growth factor) that directly drive blood vessel formation. Instead, IDO1 acts as a regulatory node between overarching inflammatory cytokines to support the maintenance of neovasculature already established within the tumor by limiting blood vessel regression.

Beyond cancer, inflammatory neovascularization is a consequential aspect of many other diseases (44). This raises the question of whether IDO1 might have a broader role supporting neovascularization in inflammatory pathologies that do not involve cancer. To begin to explore this possibility, we looked at the impact of IDO1 loss in OIR (oxygen-induced retinopathy), which is a mouse model for ROP (retinopathy of prematurity), a complication that can develop in premature infants receiving neonatal intensive care. Retinopathies are diseases of the small retinal blood vessels in which progression manifests as elevated neovascularization, (proliferative phase), and which have long been known to involve inflammatory mediators (45). OIR is a well-established model for studying the development of neovascularization as a component of eye disease (46). In the OIR model, loss of IDO1 activity, either genetically or pharmacologically, was demonstrated to significantly reduce neovascularization in a IFNγ-dependent manner. Furthermore, as observed in the lung metastasis model, loss of IL6 recapitulated the effects that IDO1 loss produced on neovascularization (38). Therefore, while the ability of IDO1 to support inflammatory neovascularization can contribute to the establishment of a tumor-promoting inflammation, it can also underpin the vascular pathophysiology of non-malignant diseases as well.

### IDO1 signals through intracellular tryptophan depletion to support neovascularization

The potential for IDO1 to enzymatically signal through both tryptophan depletion as well as production of downstream catabolites has been explored in the context of T cell tolerance. As noted earlier, while there is evidence on both sides, current opinion skews towards favoring the role of catabolites, particularly the role of kynurenine as an AHR ligand, as the principal mechanism for promoting T cell tolerance. In contrast, investigation into the underlying basis for IDO1-mediated support of neovascularization has identified activation of GCN2 and consequent triggering of the ISR in response to IDO1-mediated tryptophan depletion as the relevant underlying signaling mechanism. *Gcn2* ^-/-^ mice phenocopy the effects of both IDO1 and IL6 loss on neovascularization in both the 4T1 lung metastasis and OIR models (47). Furthermore, GCN2 signaling through the ISR is required, both *in vitro* and *in vivo*, for the IDO1 elicited induction of IL6, even while the induction IDO1 itself is unaffected by GCN2’s absence, consistent with GCN2 functioning as a downstream effector (47). The concern that IDO1 activity cannot sufficiently modify the availability of environmental tryptophan to signal through a depletion mechanism appears not to be germane in this case, as the activation of the ISR and consequent production of IL6 occur intracellularly so that IDO1 only needs to deplete internal stores of tryptophan to produce its effect. The possibility that distinct signaling mechanisms are at play, involving either tryptophan depletion or catabolites, in mediating the different biological effects of IDO1 may present opportunities for employing selective agents that can preferentially elicit a particular response.

### IDO1 expression in a discrete myeloid cell population supports neovascularization

MDSCs (myeloid-derived suppressor cells), a heterogeneous population of immature myeloid cells functionally defined by their ability to cause T cell suppression, are implicated in promoting cancer and other diseases (48). In addition to their immunosuppressive activity, MDSCs have been proposed to be pro-angiogenic (49). Initial staining for IDO1 in 4T1 metastases identified a subset of cells staining positive for the Gr1 cell surface marker, a characteristic identifier of MDSCs. However, CD11b, another defining MDSC marker, was very low or absent on the IDO1-expressing cells, intimating that these cells represent a discrete population (47). IDO1-expressing Gr1^+^ CD11b^lo^ cells were identified in both 4T1 metastases and OIR retinas in close association with endothelial cells of the neovasculature. Depleting Gr1^+^ cells in OIR neonates suppressed neovascularization, phenocopying the impact of IDO1 loss. Incorporation of IDO1-expressing Gr1^+^ CD11b^lo^ cells isolated from 4T1 metastases into a Matrigel plug engrafted under the skin demonstrated that these cells were capable of eliciting neovascularization in the plug, whereas conventional Gr1^+^ CD11b^+^ MDSCs did not exhibit any similar capability (47). The Gr1^+^ CD11b^lo^ population isolated from *Ido1* ^-/-^ , in contrast, was not able to promote Matrigel plug neovascularization when engrafted into a WT host, demonstrating that IDO1 not only identifies the subset of myeloid cells that promotes neovascularization but is required for these cells to manifest this ability. Furthermore, when an IDO1 inhibitor was administered to WT mice with Matrigel plugs incorporated with WT Gr1^+^ CD11b^lo^ cells during the final 72 hours of the experiment, neovascularization in the plug was also markedly reduced, consistent with the need for IDO1 activity to sustain neovascularization even after it has been established. Supporting the inflammatory crosstalk model, Gr1^+^ CD11b^lo^ cells isolated from either *Il6* ^-/-^ or *Gcn2* ^-/-^ mice behaved like *Ido1* ^-/-^ cells in the Matrigel assay, with diminished neovascularization relative to WT. Also in line with expectations, using *Ifng* ^-/-^ mice as hosts in the Matrigel assay restored the ability of Gr1^+^ CD11b^lo^ cells isolated from *Ido1* ^-/-^ to promote neovascularization, consistent with the induction of IDO1 by IFNγ acting as a negative feedback attenuator of IFNγ’s ability to promote neovascular regression (**Figure 1**).

MDSCs have been defined traditionally by their T cell suppressive function. Given the distinct biological capability exhibited by IDO1^+^ Gr1^+^ CD11b^lo^ cells to support neovascularization, which functionally distinguishes them from MDSCs, these cells have been designated with the moniker IDVCs (IDO1-dependent vascularizing cells, **Figure 1**) (47). Further refinement of this IDVC population revealed that neovascularizing cells were restricted to a highly autofluorescent subpopulation within which IDO1 expression was associated with strong surface staining for CD11c (associated with dendritic cells) and asialo-GM1 (associated with NK cells). These two markers divided the Gr1^+^ CD11b^lo^ AF^+^ cell population into roughly equivalent groups of (i.) CD11c^-^ asialo-GM1^-^ cells that lacked IDO1 expression and (ii.) CD11c^+^ asialo-GM1^+^ cells that appeared to uniformly express IDO1, indicating that these additional markers were sufficient to isolate the IDVC population to near homogeneity (47). This analysis also added an additional wrinkle into interpretation of the cell biology underlying IDO1’s role in inflammatory neovascularization, because all of the autofluorescent cells exhibited neovascularizing functionality, dissociating for the first time in these experiments a direct association between IDO1 and the capacity to promote neovascularization. Consistent with previous observations, when *Ido1* was genetically ablated in the IDO1-expressing CD11b^+^ asialo-GM1^+^ cells, neovascularization of Matrigel plugs was not elicited in WT mice, but only in *Ifng* ^-/-^ mice. However, IDO1-nonexpressing CD11c^-^ asialo-GM1^-^ cells isolated from *Ido1* ^-/-^ mice were equally effective at eliciting neovascularization of Matrigel plugs in WT as much as in *Ifng* ^-/-^ mice. These results drive home the conclusion that the underlying process responsible for neovascular formation is IDO1-independent. Nonetheless, IDO1-expressing IDVCs controlled the neovascularization process by engaging the IFNγ-mediated elimination mechanism, while also counterregulating this effect through IDO1-mediated induction of IL6. Furthermore, the mechanism through which IDVCs exert control over neovascularization appears to be dominant because the CD11c^-^ asialo-GM1^-^ cells from *Ido1* ^-/-^ mice were capable of sustaining Matrigel plug neovascularization in WT mice only when they were separated from the CD11c^+^ asialo-GM1^+^ cells (47).

## Discussion

Over the past 35 years following the groundbreaking proposition that tryptophan catabolism by IDO1 acts as a mechanism of T cell suppression involved in protecting the developing fetus from rejection by the maternal immune system (14), numerous studies have been directed towards elucidating the underlying basis for this immune regulatory effect and how it might be relevant to the realm of cancer therapy (50). The more recent discovery that IDO1 is also involved in maintaining inflammatory neovascularization now adds a new dimension to our understanding of how IDO1 can function as a modifier of the inflammatory milieu to render it pro-tumorigenic. Through its capacity to act as an immunometabolic enzyme that propagates a state of tumor-promoting inflammation both by enabling tumoral immune escape and sustaining tumor neovascularization, IDO1 epitomizes a quintessential, non-oncogenic tumor promoter, integrating nearly all the cancer ‘hallmarks’ that involve interfacing with the TME rather than being tumor cell-intrinsic (51).

Understanding IDO1 in this light raises the question of why such a powerful tumor-promoting metabolic enzyme, disconnected from the basic housekeeping function of maintaining tryptophan homeostasis, exists in the first place. One source of insight may come from investigations into the role of IDO1 in normal physiological inflammatory processes such as pregnancy and wound healing. Embryogenesis is an inflammatory process, which, like cancer, involves establishment of immune tolerance as well as neovascularization and vascular remodeling. The immunosuppressive role of IDO1 in protecting the hemiallogenic fetus was recognized even before its relevance to cancer was established (14). The role of IDO1 in decidual vasculaization and spiral artery remodeling has yet to be explored, however, both IFNγ and IL6 have been noted to play important roles in this process, strongly suggesting an intermediary role for IDO1. Furthermore, IDO1 deficiency can lead to symptoms associated with preeclampsia, both in mice and humans (52–54), suggesting a resultant impairment in embryonic blood vessel development. In the inflammatory process of wound healing, signaling by IL6 and IFNγ are implicated in blood vessel establishment and regression, respectively (55, 56); this again points to a possible intermediary role for IDO1 in orchestrating the phased progression of this process.

Studies focused on elucidating the observed association between IDO1 and neovascularization, have thus far led to the determination that IDO1 exerts this effect through its ability to respond to and alter the local inflammatory profile. Specifically, it has been shown that IDO1 is induced in IDVCs, a discrete population of myeloid cells, in response local IFNγ, resulting in GCN2-dependent production of IL6. Having delineated the molecular and cellular processes that are involved, questions remain regarding how this inflammatory network controls neovascularization. While IDVCs may be implicated in eliciting the IFNγ that drives this inflammatory process (47), it is clear that IDVCs are not the source of the IFNγ. Several different immune cell types have the capacity to produce IFNγ, but which specific host cells are involved in this inflammatory network remains to be determined. The Matrigel assay may provide a reductive setting in which to address this question, since only IDVCs are present in the matrix at the start which may facilitate the identification and validation of the IFNγ-producing cells that are recruited to the local environment. Additionally, there remains uncertainty regarding how IFNγ exerts its anti-neovascular effect and how IL6 functions to counteract this effect. Genetic mouse studies have concluded that IFNGR (IFNγ receptor) must be expressed on the surface of endothelial cells, (but not on hematopoietic or other stromal cells), for regression to occur (57). Downregulation of DLL4 (delta like canonical Notch ligand 4) activation of the NOTCH pathway as a consequence of engagement of IFNGR on endothelial cells has been proposed as a mechanism through which IFNγ exerts its anti-neovascular effect (58). In contrast, IFNGR signaling in non-endothelial cells had the opposite effect of promoting tumor growth, consistent with the counter-regulatory production of IL6 elicited by IFNγ-mediated induction of IDO1. How IL6 interferes with the anti-neovascular effect of IFNγ has yet to be investigated. Published work offers clues of regulatory crosstalk between IL6-activated STAT3 signaling and NOTCH signaling that may be relevant, namely that knocking down STAT3 expression abrogated MDSC-mediated activation of NOTCH (59). However, rather than a focus on neovascularization, this study focused on how IL6 and NOTCH signaling cooperate to promote cancer cell stemness, suggesting yet another possible intersectional mechanism by which IDO1 contributes to fostering a tumor-promoting inflammatory TME.

Defined functionally, IDVCs were isolated based on expression of IDO1 and the ability to elicit neovascularization in an IDO1-dependent manner within a subcutaneous Matrigel plug. MDSCs, and predominantly PMN-MDSCs comprise the vast majority of TILs (tumor infiltrating lymphocytes) present in 4T1 lung metastases (60). Iterative rounds of flow cytometry-based enrichment of IDVCs from dissociated 4T1 lung metastases established these cells as a discrete myeloid cell population separable from MDSCs by an eclectic marker profile. While IDVCs share some similarities with MDSCs, particularly the surface expression of Gr1, they are clearly distinguishable both by flow cytometry and functionality. The identification of this distinct IDVC population calls into question the entrenched concept that, in addition to their suppressive activity, MDSCs are also capable of promoting angiogenesis (49). Matrigel studies in which IDVCs were separated from MDSCs found that MDSCs exhibited no demonstrable capacity to independently elicit neovascularization (47). One caveat is that the PMN-MDSC subtype represents >80% of all immune cells in this model, so it cannot be completely ruled out that the ability of alternative MDSC subtypes, (M-MDSCs or Eo-MDSCs), to elicit neovascularization may have been missed. However, it seems more likely that MDSCs were mistakenly ascribed pro-angiogenic activity through inadvertent contamination with IDVCs. Studies establishing this angiogenic functionality have sometimes relied on the Gr1 marker alone for isolation (61), in which case IDVC contamination would be unavoidable. Even in instances in which both CD11b and Gr1 markers were both used for MDSC isolation (62), IDVC contamination appears likely given that IDVCs do express a low level of CD11b that would likely fall within the lower gating cutoff for this marker (47).

Likewise, despite reports to the contrary (63), no evidence of IDO1 expression directly in endothelial cells was detected (47). However, since IDVCs were found to be intimately associated with the endothelial neovasculature, it is conceivable this may have previously been misconstrued as endothelial expression. The IDVCs isolated from 4T1 metastases appear to be a homogeneous population with a clearly defined cell surface marker profile. Likewise, in the OIR model, IDO1-expressing cells with a corresponding marker profile have been detected immunohistochemically in the retina. Why IDVCs associate with neovascularization in these experimental models and where they originate from, either as tissue resident cells or from the circulation, are questions that have yet to be addressed. Clearly these cells are not present in all tumors, since the primary 4T1 tumors established orthotopically in the mammary fatpad exhibited no evidence of IDVC infiltration, suggesting that the local tissue environment may be a critical factor for determining whether IDVCs are involved in the TME. Also, since IDVCs are defined functionally, it has not yet been exhaustively explored whether IDVCs are restricted to the cell surface marker profile identified in these models or whether divergent cell types also exhibit IDO-dependent neovascularizing capability in other contexts.

Characterization of IDVCs thus far has been restricted to murine cells and a human equivalent has not yet been identified. Experiments with established myeloid cell lines has indicated that the signaling connection between IDO1 and IL6 operates similarly between species (47). With MDSCs, there appears to be little correspondence between the markers on human and murine cells, and if IDVCs follow this precedent, identification of a human IDVC may be challenging, requiring the development of a biological assay that can, like the mouse Matrigel assay, assess human cells for neovascularizing activity.

In different tumor settings, IDO1 expression has been detected in a variety of cell types, including tumor cells as well as in various stromal cells, (DCs, macrophages, and monocytes) (18). The determination that there are at least two distinct mechanisms, (tryptophan depletion and kynurenine production), through which IDO1 can contribute to tumor-promoting inflammation increases the likelihood that IDO1 induction within a tumor may not always have the same biological consequences. This added complexity means that therapeutic approaches should be guided by an informed analysis of the particular relevance of IDO1 expression within the tumor type being targeted because defeating immune tolerance will have different treatment implications than eliminating neovasculature. Tumors in which IDO1 is driving inflammatory neovascularization would be expected to have a vasculature that is acutely sensitive to IDO1 inhibitor treatment, with the initial response likely to be a spike in hypoxia/nutrient deprivation stress as a consequence of the rapid vascular regression mediated by IFNγ (57). Such a response might be counterproductive in the context of conventional cancer therapy since poorly vascularized, hypoxic tumors are known to be resistant to radiation and chemotherapy (64). However, several agents have been developed with the goal of specifically targeting hypoxic/nutrient deprived tumors (64), and administering an IDO1 inhibitor in conjunction with this sort of agent might elicit an enhanced combinatorial benefit.

Unlike the hemorrhagic necrosis induced by TNFα, intravital microscopy has revealed that IFNγ-mediated blood vessel regression is characterized by controlled remodeling in which first small and then progressively larger vessels disappear through a process that involves lumen collapse and vessel occlusion (57). In this manner, IFNγ-driven regression resembles non-apoptotic processes that occur during development, wound healing, and remodeling of uterine arteries during pregnancy (57). Consistent with the precise remodeling associated with these normal biological processes, IDO1 inhibitors only destroyed abnormal neovasculature, leaving established vasculature intact (38). Additionally, unlike VEGF-targeting antibodies, IDO1 inhibition did not interfere with normal revascularization. Thus, it is possible that IDO1 inhibitor treatment may ultimately lead to tumor vessel normalization, possibly providing an environment that is more amenable to conventional cancer therapy (65). Through its capacity to support neovascularization, IDO1 contributes to an inflammatory state that not only is tumor promoting, but also more broadly pathogenic. In the OIR model, IDO1 is induced by IFNγ during the inflammatory response to ischemic stress, and signals to IL6 to maintain the pathologic neovascularization that underlies disease complications, in line with inflammation being the underlying driver of the transition from a benign, non-proliferative disease state to a harmful, proliferative disease state. In human diabetic retinopathy patients, an association between IDO1 expression and disease progression has been noted (66), which coincides with an upregulation of IL6 (67) consistent with the possibility that inflammatory misalignment by IDO1 may be a contributing factor to this disease.

When first proposed, the concept that a catabolic enzyme like IDO1 could be involved in the regulation of the higher order process of immune function was considered a rather fringe idea. Now myriad such regulatory interfaces are recognized, with the emerging field of metabolomics driving the large-scale study of small-molecules and their interactions with biological systems. Still, the role that IDO1 plays in shaping tumor-promoting inflammation appears to be uniquely consequential, raising the question Why IDO1? In particular, the ISR is responsive to a wide range of inputs, and the ability of IDO1 to signal through this pathway is not unique. Perhaps a contributing factor is IDO1’s functional redundancy among tryptophan catabolizing enzymes, freeing IDO1 to assume these broader regulatory functions. TDO2 and TPH (tryptophan hydroxylase) are responsible for the basic biochemical processes of initiating tryptophan catabolism down the kynurenine and serotonin pathways, respectively. There is also a third enzyme, IDO2, which is related to IDO1 through gene duplication and which phylogenetic analysis has identified as being more aligned with the ancestral gene (68). While the biological function of IDO2 is not yet entirely clear, and may not even fully involve tryptophan catabolism (69), IDO1, as the duplicate, would again be superfluous to this function. Accordingly, it is less a unique aspect of IDO1 function that dictates its ability to foster tumor-promoting inflammation so much as a unique set of regulatory controls that has been superimposed because IDO1’s activity is not otherwise needed. This line of reasoning may inform expectations regarding the involvement of IDO1 in different tumor contexts. For a tumor that develops in the absence of IDO1-associated inflammation, the selective pressure exerted by immunoediting would still select for elevated tryptophan catabolism as a component of the escape process. However, this would not necessarily lead to the upregulation of IDO1, as TDO2 or the broader amino acid catabolizing enzyme IL4I1 (interleukin 4 induced 1) (70) might be engaged. Such an outcome is consistent with the involvement that has been reported for these alternative enzymes in some tumor types (71). Conversely, in the context of an IDO1-inducing, inflammatory environment, it is likely that the tumor will become specifically reliant on IDO1 in a manner analogous to the concept of ‘oncogene addiction’ (72, 73). Understanding the involvement of IDO1-orchestrated tumor promoting inflammation in specific tumor types may thus be consequential for predicting whether they will be more amenable to IDO1-specific inhibitors or whether more broad-based pan-inhibitors will be required for successful intervention. The lack of an increased survival benefit obtained with the small molecule IDO1 inhibitor epacadostat when combined with the PD1 blocking antibody pembrolizumab in a Phase 3 trial for unresectable or metastatic melanoma (74) was a stark reminder that successful clinical development of an effective cancer drug is hardly a sure thing (75). Continued preclinical investigation into how IDO1, and tryptophan catabolism more generally, contribute to tumor development in different settings will be critical to developing the more sophisticated contextual framework essential to informing future development of IDO1 inhibitors for cancer treatment.

## Author contributions

AJM wrote the first draft of the manuscript. GP wrote sections of the manuscript. AM and SD performed the bulk of the experimental work reviewed in the manuscript. All authors contributed to the article and approved the submitted version.
